# Complexities and similarities of HLA antigen distribution in Asian subcontinent

**DOI:** 10.4103/0971-6866.73397

**Published:** 2010

**Authors:** U. Shankarkumar

**Affiliations:** Scientist “D”, National Institute of Immunohaematology, 13^th^ Floor, K.E.M. Hospital, Parel, Mumbai – 400 012, India

Human population has increased from 3,031,092,442 in 1960 to 6,879,100,100 as on date. The highest populated country of the world is China (19.5%) followed by India (17.3%). Population genetic frequencies are ethnically based on the religion, country, region and community that are studied. Some of the major ethnic groups as per the region of the world are American Indians, Australoid Aboriginals, Caucasoid, Hispanics, Orientals, Blacks, Pacific Islanders, Iranians, Negroids, Persians, Russians, Jews etc., depending on the continents such as Africa, America, Asia, Australia. Many religious linguistic groups in each part of the world have evolved during earth’s time course as rightly pointed out in 1973[1] that “one of the greatest experiments of Nature was the Caste system in India”. HLA system, the most polymorphic and complex set of genetic markers known in man, is of valuable significance in anthropological, immunological and clinical studies.[2] These cell-surface antigens play an important role in the activation of immune-competent cells, thereby promoting immune response. The extensive polymorphism of the HLA system is associated with a large peptide repertoire for initiating immune responses against a wide range of foreign antigens (pathogens). Despite the fact that human population went through a constriction >150 kya, that was capable of fixing many loci, the HLA loci appear to have survived such a constriction with great deal of variation. As on date there are 5674 HLA alleles detected. The most variable alleles are found in the HLA B and HLA DRB1 loci.

India, with 1,189,700,000 people, having 3824 castes and 461 tribes and many unique languages families, is one of the megadiversity countries and the largest democracy in the world. It is the second continent to have been occupied by man since the past 60,000 years. Many early migrations settled in various parts of the world especially Central Asia, Eurasia, Middle East, Pakistan and India when huge expansions of human occurred. As a result, almost all forms of religious and social events like marriage and family styles have been experimented. The basic pattern of the society and value system seems to have been laid down well before the origin and spread of Dravidian and other languages in India. While India was an ancient land of immigration spread of very many streams of people, Africa was a land of origin, expansion and divergence of the same gene pool for the past 0.2 million years.

A caste or tribe is a breeding unit and a breeding isolate: as time passes they drift away from one another. A tribe in most of the instances are primitive in their mode of subsistence, economy and living conditions, mostly living in isolation in hilly terrains sight of the modern developments. Castes on the contrary live in plains and are capable of articulation and egging their income in more modern interdependent societies, villages and township. Most of these castes and tribes in India are inbred and endogamous, though the degree varies from one region to other. Each caste/tribe is made of many clans, mostly patriliny, though matriliny is the ancient form of lifestyle and is practised even today in many tribes of India. Each caste/tribe is a social unit and a social security system defined by their own characteristics, territory, space, job and interdependency. Different castes living in the same region, sharing the environment and epidemiology as on date are sympatrically isolated in terms of their gene pool. This has great significance in terms of epidemiology and infectious disease transmission and susceptibility. It is known that not all the infected develop the disease.[3]

People having two different genetic make up may not be equally susceptible to a given disease, though the nature–nurture interaction plays a dominant role in the incidence and prevalence of various diseases. Theoretically, high polymorphism of HLA gene can occur due to mutation rate, selection, genetic hitchhiking or a combination of all the three. Indigenous populations or caste/tribal groups show a very restricted diversity of alleles at a particular HLA locus consistent within a population. Studies on HLA allelic diversity of among 838 population groups from Asia (272), Western Europe (147), South and Central America (107), North America (79), Pacific (59), sub-Saharan Africa (55), Eastern Europe (56), Middle East (34), North Africa (21) and Australia (8) of the world have revealed that some common alleles such as HLA A*02 (20–28%), B*40 (5–20%), and DRB1*15 (10–18%) are seen in Asian countries such as Afghanistan, Armenia, Azerbaijan, Bahrain, Bangladesh, Bhutan, Brunei Darussalam, Cambodia, China, East Timor, Georgia, India, Indonesia, Iran, Iraq, Israel, Japan, Jordan, Kazakhstan, Lebanon, Malaysia, Maldives, Middle East, Mongolia, Myanmar, Nepal, North Korea, Oman, Pakistan, Philippines, Qatar, Saudi Arabia, Singapore, South Korea, Srilanka, Syria, Taiwan, Tajikistan, Thailand, Turkmenistan, UAE, Uzbekistan, Vietnam and Yemen.[4] Specific HLA alleles and their associated haplotypes were found uniquely in a particular population group in high frequency. Earlier studies from India have revealed high frequencies of HLA B48 among Patel’s, B14 among Parsees and Badaga tribes, B21 among Koya tribe etc.[5] Further HLA molecular techniques advancement has redefined the HLA allele subtypes that have resulted in identification of new HLA alleles and novel HLA haplotypes in many populations studied from Asia. HLA A*02, B*40 and DRB1*15 are the common alleles. HLA A*02 has 282 molecular subtypes, HLA B*40 has 158 molecular subtypes and HLA DRB1*15 has 51 molecular subtypes as on date. It has been reported in literature that HLA A*02:01:01:01, A*02:03:01, A*02:05:01, A*02:06:01, A*02:07, A*02:09, A*02:11, A*02:22:01 and A*02:36:01 and their related haplotypes from India.[6] HLA haplotypes identified with relation to A*02 allele were A*02-B*51, A*02-B*52, A*02-B*08, A*02-B*13, A*02-B*51, A*02-B*14, A*02-B*15, A*02-B*44, A*02-B*51, A*02-B*27, A*02-B*18, A*02-B*35 A*02-B*37, and A*02-B*40. Further HLA A*02-B*40-DRB1*15 haplotype has been reported in high frequency in Pakistan and HLA A*02:11 B* 40:06:01:01 DRB1*15:01:01 in India. Novel alleles such as A*33:06, B*27:08, B*27:14, DRB1*15:08 along with high-frequency alleles such as A*02:11, A*33:03:01, B*27:05:01 have also been reported.[7] Report of HLA allele distribution among Pakistan population in this issue reveals that the common alleles were A*02, B*35 and Cw*07 among HLA Class I while DRB1*03, DRB1*07, DRB1*11 and DRB1*15 and 179 different as well as 285 unique haplotypes along with the common Asian class II haplotype DRB1*15-DQB1*06, which have been reported in many of the other oriental populations.[8] It is suggested that these allele may have been generated by point mutation or gene conversion from the ancestral allele after the group separated from the other group. Multiple polymorphic alleles in an indigenous population group are maintained at appreciable frequencies due to over dominance (heterozygous advantage), frequency-dependent selection, bottleneck effect or other selective forces.[2] Both selective forces and a high rate of germline diversification are involved in the evolution of HLA allelic diversity. The implications of these polymorphic diversities are important in community genetics in general. HLA-A, HLA-B and HLA-DR have long been known as major transplantation antigens. Recent clinical data indicate that HLA allele matching also affects the clinical outcomes of hematopoietic stem cell transplantation. Common HLA haplotypes need to be studied in order to identify matched unrelated donors for bone marrow transplantation.[9] Therefore studies on the HLA allelic and haplotype diversity among the Asian populations such as Pakistan reported in this issue will allow us to know the common alleles and their associated haplotypes in order to identify an unrelated compatible donor in every transplant centers in Asian countries. HLA diversity of a population is not only a theoretical exercise or an exercise to peep into the past history of the country’s genetic endowment and how it came into being. Its implication in finding related/unrelated donors for bone marrow/solid organ transplantation, its association with various diseases, its clinical utility in understanding drug reactions (antiretroviral, antiepileptic etc) and its overall biological relevance to adaptive immunity and vaccine development implores that we need much more extensive HLA data generated from many of the Asian countries including Pakistan. The paper under discussion in spite of all its technical short comings is a valuable welcome addition to the scientific literature.
